# Editorial: Innovative multidisciplinary insights into the gut-liver axis and cancer

**DOI:** 10.3389/fonc.2025.1588708

**Published:** 2025-04-22

**Authors:** Rohit Gundamaraju, Bey Hing Goh, Rajeev K. Singla

**Affiliations:** ^1^ ER Stress and Mucosal Immunology Lab, School of Health Sciences, University of Tasmania, Launceston, TAS, Australia; ^2^ Department of Pharmaceutical Engineering, B V Raju Institute of Technology, Medak, Telangana, India; ^3^ Sunway Biofunctional Molecules Discovery Centre, Sunway Medical & Life Sciences, Sunway University, Petaling Jaya, Selangor Darul Ehsan, Malaysia; ^4^ Faculty of Health, Australian Research Centre in Complementary and Integrative Medicine, University of Technology Sydney, Ultimo, NSW, Australia; ^5^ Department of Pharmacy and Institutes for Systems Genetics, Center for High Altitude Medicine, Frontiers Science Center for Disease-related Molecular Network, West China Hospital, Sichuan University, Chengdu, Sichuan, China; ^6^ School of Pharmaceutical Sciences, Lovely Professional University, Phagwara, Punjab, India

**Keywords:** tumor microenvironment, gut microbiota, cancer, gut-liver axis, innovative interventions

Over time, the tumor microenvironment has been found to display an affinity for phenotypic and functional diversity that is now said to be altered by microbes. Recently, the microbiota has been reported to be instrumental in coping with environmental stress and to influence interactions with the host (1). The axis between the intestine and liver has been connected to bile acids, and some pathophysiological conditions induce DNA damage and trigger a carcinogenic environment. Furthermore, there has been evidence of a bidirectional pathway that creates an independent environment for itself (2). Several findings support the role of gut microbiota in liver cancer therapy. Microbiota-mediated immune alterations determine anti-tumor responses in hepatobiliary cancer (3).

In murine studies of a compromised gut barrier, introducing microbially influenced compounds into the circulation led to liver cancer initiation (4, 5). Interestingly, cancer signaling from these immunocompromised intestines was observed in chemically induced hepatocarcinoma (5). Evidence for the triggering of senescence aging in hepatocarcinoma by gut microbiota strengthened the theory of the gut-liver axis (6). The Research Topic includes pertinent articles to understand the complexities between the gut and the liver, focusing on the microbiota and how it can shape different approaches. The manuscripts published in the Research Topic support the above concepts, which would help us to better understand bidirectional relationships. An aggregate of four high-quality review publications was showcased in this Research Topic. These manuscripts underpin the complexity of the gut microbiota and how it influences a series of events in the hepato-gastrointestinal tract.

A study by Xu et al. elucidated the importance of the gut microbiota and its ability to induce hepatic encephalopathy and how post-TIPS HE transjugular intrahepatic portosystemic shunt hepatic encephalopathy can be influenced by gut microbiota-mediated treatment strategies.

Zheng et al. highlighted the effect of intratumoral microbiota in shaping tumor initiation and development and how it can orchestrate the tumor microenvironment. The review also discusses microbiota-mediated cancer signaling. The manuscript aims to decipher how tailored bacteria can target tumors and how intratumoral microbiota can best be employed to enhance therapeutic potential and impede inflammation. The review showcases the possibility of considering microbes as an anti-cancer therapy.

A state-of-the-art review by Yang summarized the intricate relationship between the microbial flora and the tumor microenvironment and how it establishes the fate of the tumor progression. The bacterial extracellular vesicles in symbiosis with the microbiota have been shown to augment chemotherapy. The review explicitly identified the targets of intestinal microbes and how they can control the tumor microenvironment, which will be regarded as fundamental for novel therapeutics.

Wang et al., in their review, discussed the possibility of enhancing therapeutic response with the aid of the diet-microbiota axis. Since diet is considered a modifiable factor, manipulating the gut microbiota with a specific diet is believed to possess a beneficial role, and beneficial microbes may mitigate the deleterious effects of cancer. The authors state that microbiota can officially be employed as an adjuvant therapy and summarize all the previously published gut-microbiota-mediated therapeutic mechanisms.

We are sincerely thankful to all the authors for contributing their articles, which works will further enrich the knowledge in this field and will be helpful for the readers.
